# Hypercalcemia of Malignancy and Medication-Related Osteonecrosis of the Jaw Following Denosumab Discontinuation in Metastatic Breast Cancer: A Case Report

**DOI:** 10.7759/cureus.91082

**Published:** 2025-08-27

**Authors:** Omar Shazley, Christina Orr

**Affiliations:** 1 Basic Sciences, Saint James School of Medicine, Kingstown, VCT; 2 Diabetes and Metabolism Endocrinology, Vancouver Clinic, Vancouver, USA

**Keywords:** medication-related osteonecrosis of the jaws (mronj), metastatic breast cancer (mbc), non-pth-related hypercalcemia of malignancy, severe hypercalcemia, zolendronic acid

## Abstract

Hypercalcemia of malignancy (HCM) is a common and serious complication in patients with advanced cancer and is associated with significant morbidity and mortality. Standard long-term management of HCM relies on anti-resorptive therapies such as bisphosphonates and denosumab, both of which act by inhibiting osteoclast-mediated bone resorption but carry the risk of medication-related osteonecrosis of the jaw (MRONJ). This case illustrates a patient with metastatic breast cancer (MBC) who developed recurrent, severe hypercalcemia after discontinuing denosumab due to MRONJ, followed by profound hypocalcemia upon the initiation of zoledronic acid. This case presentation signifies the challenges of maintaining calcium homeostasis in interrupted anti-resorptive therapy and the risk of rebound hypercalcemia after denosumab withdrawal. Continued monitoring to manage calcium disturbances and skeletal complications is required in cancer patients at risk for or experiencing MRONJ.

## Introduction

Hypercalcemia of malignancy (HCM) is a frequent and severe complication in patients with advanced cancers, particularly those with bone metastases such as metastatic breast cancer (MBC). The underlying mechanisms of HCM are diverse and include elevated parathyroid hormone (PTH)-related protein (PTHrP), cytokine-driven local osteolysis, increased tumoral production of 1,25-dihydroxyvitamin D, and, less commonly, ectopic secretion of PTH. To assess etiology, a standard diagnostic approach includes evaluating serum calcium, PTH intact (PTH-I), PTHrP, and vitamin D metabolites.

The bone is considered a common metastasis site for breast cancer, with up to 75% of patients developing bone involvement during the disease [1]. The association between metastatic tumor cells and the bone microenvironment initiates a self-perpetuating cycle as tumor cells promote osteoclast-driving bone resorption, which releases calcium and growth factors that promote further tumor growth and bone destruction [1]. This process not only elevates the risk of skeletal complications, including fractures and spinal cord compression, but also impairs calcium regulation, which frequently culminates in HCM [1]. Calcium ions released from bone during metastasis act as key signaling molecules, exacerbating tumor growth and bone resorption and highlighting the central role of calcium regulation in the pathophysiology of bone MBC [1].

To mitigate skeletal complications and control calcium levels, anti-resorptive agents, including Xgeva (denosumab), a RANKL inhibitor, and Reclast (zoledronic acid), a bisphosphonate, are standard therapies in patients with bone metastases from breast cancer [2]. These agents suppress osteoclast-mediated bone resorption, decreasing the risk of fractures and malignant hypercalcemia [2]. Both treatments, however, carry a risk of medication-related osteonecrosis of the jaw (MRONJ), a condition characterized by exposed, non-healing bone in the jaw often initiated by dental procedures or pre-existing oral diseases. The risk of MRONJ increases with cumulative exposure to anti-resorptive therapy and is notably higher with denosumab compared to zoledronic acid [3-5]. Studies have reported incidence rates of MRONJ ranging from 1% to 17%, with higher rates observed in patients receiving denosumab and in those with prolonged therapy duration or pre-existing oral disease [4,5].

Discontinuation of anti-resorptive therapy can precipitate marked disturbances in calcium metabolism. Withdrawal of denosumab, especially in patients with high tumor burden in bone, can lead to rebound hypercalcemia due to unopposed osteoclast activity and excessive bone resorption [3-6]. This phenomenon is well-documented and can result in severe, sometimes life-threatening, hypercalcemia requiring urgent intervention [1,6]. Conversely, initiation or re-initiation of bisphosphonate therapy, such as zoledronic acid, can cause acute hypocalcemia, typically in patients with predisposing risk factors, including vitamin D deficiency or hypoparathyroidism [7].

This case presents a patient with MBC who developed severe hypercalcemia after Xgeva (denosumab) discontinuation, subsequent hypocalcemia with Reclast (zoledronic acid), and MRONJ, highlighting the complexity of calcium management and anti-resorptive therapy in advanced malignancy.

## Case presentation

A 67-year-old woman with a complex oncologic and endocrine history was diagnosed with MBC in June 2012 after presenting with chest and shoulder pain. Imaging at that time revealed a left breast mass, left axillary lymphadenopathy, and multiple osseous metastases. Biopsies confirmed a grade 2 infiltrating ductal carcinoma of the left breast, which was strongly estrogen receptor positive (ER > 95%), focally progesterone receptor positive (PR 2%-5%), and HER2 3+ with a Ki-67 of 30%. The right breast biopsy showed ductal carcinoma in situ. Further staging identified metastatic involvement of the sternum, spine, and iliac crest. Her medical history was notable for Hodgkin lymphoma (status post autologous stem cell transplant), papillary thyroid cancer (status post thyroidectomy and radioactive iodine ablation), postsurgical hypoparathyroidism, hypothyroidism, multiple myelomas, kidney stones, hypertension, and prior surgical interventions including bilateral mastectomy and breast reconstruction.

Her initial management included a bilateral mastectomy and systemic therapies tailored to her evolving disease status. She started on letrozole 2.5 mg daily in June 2012, with trastuzumab (Herceptin) added in July 2012 for HER2-positive disease. Over the subsequent years, her regimen was adjusted to include pertuzumab during periods of disease progression and fulvestrant for further endocrine blockade, and later, Kadcyla (T-DM1) and trastuzumab-deruxtecan as newer HER2-targeted agents became indicated. Throughout this period, she received regular intravenous (IV) chemotherapy and endocrine therapy, with adjustments made in response to disease progression, cardiac function, and treatment tolerability.

Given her extensive bone metastases, she started on Xgeva (denosumab) 120 mg monthly in May 2013 to reduce the risk of skeletal-related events and control HCM. Denosumab was continued as her primary anti-resorptive agent through the end of 2024. Her laboratory values remained stable and within normal reference ranges during this period. Serum calcium ranged from 8.7 to 9.6 mg/dL (8.2-10.3 mg/dL), PTH was typically suppressed or low-normal from 10 to 30 (19-88 pg/mL), and PTHrP was less than 3.4 pmol/L (0-3.4 pmol/L). Her 25-hydroxyvitamin D was maintained at 35-50 ng/mL (30-80 ng/mL). Tumor markers carcinoembryonic antigen (CEA), cancer antigen 27-29 (CA 27-29), and cancer antigen, breast 15-3 (CA 15-3) were monitored and remained within or near reference limits, with CEA less than 3.0 ng/mL, CA 27-29 less than 39.0 U/mL, and CA 15-3 less than 31 U/mL (see Table 1).

**Table 1:** Treatment course before case presentation ªNot applicable Ca: calcium; PTH: parathyroid hormone; PTHrP: PTH-related protein; Vit D-25: 25-hydroxyvitamin D; CEA: carcinoembryonic antigen; CA 27-29: cancer antigen 27-29; CA 15-3: cancer antigen, breast 15-3

Date	Treatment course	Ca (8.6-10.4 mg/dL)	PTH (16-77 pg/mL)	PTHrP (0.0-3.4 pmol/L)	Vit D-25 (30-100 ng/mL)	CEA (≤2.5 ng/mL)	CA 27-29 (≤39 U/mL)	CA 15-3 (0-31 U/mL)
6/2012	Daily letrozole initiated	9.4	-	-	-	-	-	-
7/2012	Trastuzumab (Herceptin) anti-HER2 therapy initiated	-	-	-	-	-	-	-
5/2013	Palliative pertuzumab started, docetaxel (toxicity)	-	-	-	-	-	-	-
6/2013	-^a^	8.9	-	-	-	-	-	-
9/2013	Monthly denosumab (Xgeva) started	-	-	-	-	-	-	-
1/2014	-	9	-	-	-	3	-	-
6/2014	Discontinued trastuzumab & pertuzumab	-	-	-	-	-	-	-
8/2014	-	8.9	-	-	-	-	-	-
9/2014	Monthly fulvestrant started	9.3	-	-	-	-	-	-
11/2014	-	8.5	-	-	-	-	-	-
8/2015	-	8.9	-	-	-	-	-	-
12/2016	Ado-trastuzumab emtansine (T-DM1) initiated	-	-	-	-	-	-	-
9/2017	-	9.1	-	-	-	-	-	-
3/2020	-	9	-	-	-	-	34.5	-
9/2021	T-DM1 and fulvestrant stopped	8.5	-	-	-	-	358.5	-
10/2021	Trastuzumab deruxtecan (Enhertu) started	9.1	-	-	-	231.8	437.4	295
12/2022	Trastuzumab deruxtecan 4.4 mg/kg	9	-	-	-	1.6	19.1	18
3/2023	Trastuzumab deruxtecan 3.2 mg/kg	8.7	-	-	-	1.9	18.9	19
12/2024	-	9	-	-	-	13	124.6	76

During the latter portion of this treatment course, the patient developed persistent dental and jaw symptoms, including a suppurative discharge from a sinus tract near tooth #19 that recurred over several months. Despite intermittent improvement with antibiotic beads placed by her dentist, the suppuration would return. A cone-beam computed tomography (CBCT) revealed bone loss near tooth #19. However, the CBCT imaging could not be retrieved and displayed in this report, as the study was performed at an outside institution and was inaccessible through our electronic health record system. The diagnosis for MRONJ was supported by her long-lasting symptoms, characteristic imaging findings, and the presence of necrotic bone that had not responded to continued dental care. The patient included a history of metastatic cancer involving the jaw and had been receiving long-term Xgeva for bone loss due to her cancer treatments, further increasing her risk for MRONJ. MRONJ was confirmed after consultation with her oral surgeon. She expressed uncertainty about continuing denosumab in light of her jaw complications. Denosumab was discontinued at the beginning of January 2025, marking the end of a period of stable calcium homeostasis.

Diagnostic assessment

On initial presentation on May 7, 2025, outpatient testing revealed a serum calcium of 14.5 mg/dL, which was confirmed in the emergency department (ED) with a calcium of 13.5 mg/dL. Her ionized calcium was elevated at 1.68 mmol/L. PTH and PTH-I were suppressed at 2 pg/mL. Calcium was elevated at 10.6 mg/dL. PTHrP was within normal limits at <2.0 pmol/L, and her 25-hydroxyvitamin D was normal at 68.9 ng/mL. Following initial management (discussed below in Treatment), her calcium improved to 11.3 mg/dL after one day.

She was re-hospitalized on May 20, 2025, due to recurrent episodes of hypercalcemia. Her serum calcium level was 13.0 mg/dL, followed by persistently high ionized calcium at 1.68 mmol/L. Her PTH level was below the normal range at 6.0 pmg/mL. Her 25-hydroxyvitamin D level was suppressed at 10 ng/mL. PTHrP was consistent within normal limits, supporting a non-parathyroid, non-endocrine mechanism for her hypercalcemia. Additional workup revealed a normal kappa/lambda ratio and the absence of an M spike on serum protein electrophoresis, making multiple myeloma unlikely. Her baseline creatinine was approximately 1.15 mg/dL, but on admission, her blood urea nitrogen (BUN)/creatine ratio was 35, likely reflecting hypercalcemia-induced dehydration and decreased oral intake, further exacerbated by the concurrent use of angiotensin receptor blocker (ARB) and spironolactone (see Table 2).

**Table 2:** Treatment course during case presentation ªNot applicable Ca: calcium; Ca (ionized): calcium (ionized Bld gas); PTH: parathyroid hormone; PTHrP: PTH-related protein; Vit D-25: 25-hydroxyvitamin D; CEA: carcinoembryonic antigen; CA 27-29: cancer antigen 27-29; CA 15-3: cancer antigen, breast 15-3; MRONJ: medication-related osteonecrosis of the jaw; AKI: acute kidney injury

Date	Treatment course	Ca (8.6-10.4 mg/dL)	Ca (ionized) (1.12-1.26 mmol/L)	PTH (16-77 pg/mL)	PTHrP (0.0-3.4 pmol/L)	Vit D-25 (30-100 ng/mL)	CEA (≤2.5 ng/mL)	CA 27-29 (≤39 U/mL)	CA 15-3 (0-31 U/mL)
01/01/2025	Denosumab withheld due to MRONJ	-	-	-	-	-	15.3	145.9	83
01/10/2025	Ongoing chemotherapy	9.8	-	-	-	-	21.2	193	102
02/18/2025	-^a^	9.3	-	-	-	-	26.6	195.6	120
03/11/2025	-	9	-	-	-	-	30.2	240.4	134
04/01/2025	-	9.2	-	-	-	-	40.1	309.9	156
04/21/2025	-	10.4	-	-	-	-	40.3	278.3	165
05/07/2025	Admitted for hypercalcemia	10.6-14.1	1.68	2	<2	68.9	-	-	-
05/16/2025	-	9.9	-	-	-	-	-	-	-
05/20/2025	Admitted for recurrent hypercalcemia with AKI	13.0-13.3	1.68	<6.0	-	<10	-	-	-
05/29/2025	Zoledronic acid initiated	10.9-12.6	-	-	-	-	-	-	-
06/02/2025	Admitted for hypocalcemia and hypophosphatemia	8.1	1.04	-	-	-	-	-	-
06/03/2025	Discharged	7.3	0.99	-	-	-	53.8	432.6	219
06/05/2025	-	6.9	0.99	-	-	-	-	-	-
06/06/2025	Admitted for recurrent hypocalcemia	7.2-7.5	0.99-1.01	-	-	-	5.9	-	238
06/07/2025	Discharged	7.5	1.06	-	-	-	-	-	-
06/10/2025	-	8.2	-	-	-	-	-	-	-
06/16/2025	-	9.3	-	-	30	81	-	-	-
07/01/2025	Persistent mild hypocalcemia, ongoing monitoring	9.3	-	-	-	-	68	-	-

Treatment

After initial diagnostic workup and stabilization during both hospital admissions, the patient’s management focused on safely correcting her hypercalcemia and addressing the associated metabolic disturbances. During her first hospitalization, IV fluids and calcitonin were administered, reducing her serum calcium from 13.5 mg/dL on admission to 11.3 mg/dL before discharge. She was advised to continue vitamin D supplementation at 1,000 units daily, while both calcitriol 0.5 mcg and calcium carbonate 200 mg daily were discontinued. Electrolyte repletion was also undertaken to correct potassium, magnesium, and phosphate deficiencies.

During her second admission for recurrent hypercalcemia and acute kidney injury, the patient was placed on IV fluids, Lactated Ringer’s solution, and normal saline, along with calcitonin. This effectively normalized her calcium levels. In light of her persistently suppressed PTH and the non-endocrine mechanism underlying her hypercalcemia, both calcium supplements and calcitriol remained discontinued. At discharge, she was prescribed calcitonin nasal spray (200 units per actuation) and instructed to continue daily vitamin D at 1,000 units. Her treatment plan was coordinated among oncology, endocrinology, and supportive care teams, with ongoing monitoring and adjustments as clinically indicated in the context of her MBC and recent discontinuation of denosumab due to MRONJ.

Outcome and follow-up

After her hospitalization for severe hypercalcemia in May 2025, the patient’s clinical course shifted toward hypocalcemia management. She was consequently hospitalized twice in early June (see Table 2) due to hypocalcemia and hypophosphatemia. Zoledronic acid (Zometa) was given on May 29, 2025, for the treatment of her hypercalcemia. This treatment had been approved by oral surgery following an interval of improvement in her MRONJ. She was readmitted on June 2, 2025, and presented with hypocalcemia and hypophosphatemia. Laboratory results at the time showed a serum calcium of 8.1 mg/dL and an ionized calcium of 1.04 mmol/L. Additional findings included mild leukopenia and moderate anemia. Her serum creatinine was measured at 1.43 mg/dL, consistent with her baseline renal function. She received IV sodium phosphate and phosphate infusions during this admission, gradually correcting her electrolyte abnormalities. She was discharged on vitamin D3 1,000 IU daily and calcitonin nasal spray (200 units/actuation), discontinuing calcitriol.

The patient was admitted on June 6, 2025, with symptoms of lip tingling and numbness in her fingers. Laboratory studies again demonstrated hypocalcemia, with a serum calcium of 7.2 mg/dL and ionized calcium of 0.99 mmol/L. She had discontinued calcitonin approximately eight days prior, as per outpatient instructions. Her symptoms improved in the ED following IV calcium gluconate, with a modest increase in ionized calcium. On admission, she received additional IV calcium and sodium phosphate. Her home regimen was adjusted to include magnesium oxide 400 mg twice daily, a reduced dose of calcitriol (0.25 mcg daily), calcium 200 mg supplement (equivalent to calcium carbonate 500 mg or calcium citrate 1,000 mg daily), and continued vitamin D 25 mcg daily. Calcitonin nasal spray was discontinued. Her hypocalcemia was attributed to recent zoledronic acid administration, a known complication of Zometa/Reclast infusions, particularly in patients with metastatic bone disease and prior anti-resorptive therapy.

The patient remains on trastuzumab-deruxtecan 3.2 mg/g and letrozole 2.5 mg for MBC, with Zometa continued as her anti-resorptive agent, replacing calcitonin. She was closely followed by oncology and endocrinology, with regular outpatient visits. Her calcium levels were stable at her follow-up visits, and she reported symptomatic improvement on the adjusted supplementation regimen. At the time of her latest follow-up, the patient continued with calcitriol 0.25 mcg daily, calcium 200 mg supplement, and vitamin D 25 mcg with regular oncology and endocrinology follow-up to ensure continued metabolic stability and to monitor further complications.

## Discussion

The management of HCM in patients with MBC and bone metastasis is complex, particularly when complicated by MRONJ between anti-resorptive treatments. This case illustrates the challenges of maintaining stable calcium levels in the context of advanced malignancy with bone disease and the side effects of bone treatment.

HCM is a well-established complication associated with morbidity in patients with cancer [8]. The development of HCM is multifactorial in breast cancer, influenced by direct bone destruction from metastases, elevated osteoclast activity, and, in some cases, the influence of circulating factors, including PTHrP [8]. Denosumab, a monoclonal antibody targeting RANKL, has emerged as a highly effective agent for reducing osteoclast-mediated bone resorption and controlling hypercalcemia in this population [2,8]. However, abrupt discontinuation of denosumab can result in a rebound increase in bone turnover, leading to severe, non-PTH-dependent hypercalcemia, as observed in this patient [6,9-11]. This phenomenon is increasingly recognized in both pediatric and adult populations, with the risk heightened in those with high baseline bone turnover or extensive skeletal disease [9,10,12]. For this case, the patient developed severe hypercalcemia following denosumab cessation, with laboratory findings of suppressed PTH, normal PTHrP, and normal vitamin D, effectively ruling out primary hyperparathyroidism, humoral hypercalcemia, and vitamin D toxicity as primary etiologies. The temporal withdrawal of denosumab with the absence of underlying causes supports a diagnosis of rebound hypercalcemia.

The onset of MRONJ prompted the patient to discontinue denosumab, thus further adding complexity to her management. The adverse effect of MRONJ is linked to high-dose anti-resorptive therapy, particularly in individuals with metastatic bone disease [5,13,14]. Multiple studies have demonstrated that denosumab carries a higher risk of MRONJ compared to zoledronic acid, with reportedly elevated hazard ratios and cumulative incidence rates with prolonged therapy duration [4,5,13,14]. Dental procedures with pre-existing oral disease and cumulative exposure to anti-resorptive agents amplify the risk [5,13,15]. Clinical guidelines highlight the importance of a thorough dental evaluation before considering anti-resorptive therapy for prevention and management. The interruption of anti-resorptive therapy due to MRONJ, while necessary for jaw healing, exposes patients to the risk of rapid bone resorption and metabolic instability, as demonstrated in this case. The subsequent initiation of zoledronic acid is itself associated with the risk of acute and prolonged hypocalcemia, particularly in patients with prior anti-resorptive exposure, vitamin D deficiency, or underlying endocrine dysfunction [7,16,17]. The patient’s swift change from hypercalcemia to hypocalcemia after receiving zoledronic acid signifies the importance of close biochemical monitoring and supplementation strategies when shifting between therapies [7,16,18].

The prevailing consensus among the multidisciplinary care team was that the patient’s recurrent hypercalcemia was precipitated by the discontinuation of denosumab, which had been held due to the development of MRONJ. Denosumab has been shown to confer a higher risk of MRONJ compared to zoledronic acid, with incidence rates reported between 0.5% and 3.2% after one to three years of therapy and hazard ratios ranging from 2.3 to nearly 3 in comparison to bisphosphonates [5,13,14]. The diagnosis of MRONJ was substantiated by the patient’s persistent supportive discharge from a sinus tract near tooth #19, intermittent response to local antibiotic therapy, and characteristic findings on CBCT, including osteolytic changes, interval development of osteosclerosis, and lingual plate fenestration, radiographic features well described in the literature as indicative of MRONJ progression and healing [19,20]. The necessary interruption of anti-resorptive therapy for jaw healing resulted in the loss of osteoclast inhibition, predisposing the patient to rebound hypercalcemia, a phenomenon increasingly recognized following denosumab withdrawal, particularly in individuals with a high skeletal tumor burden or prolonged exposure to anti-resorptive agents [6,9,10]. This presentation signifies the iatrogenic nature of MRONJ in the setting of potent anti-resorptive therapy for metastatic bone disease. It highlights the critical importance of careful planning when transitioning or interrupting bone-targeted agents in high-risk oncology patients.

The diagnostic workup in this case was comprehensive, excluding alternative causes of calcium disturbance such as multiple myeloma, primary hyperparathyroidism, and vitamin D toxicity. In conjunction with the clinical context, the suppressed PTH and normal PTHrP levels were consistent with malignancy-associated, non-endocrine hypercalcemia following denosumab withdrawal [8,9]. The subsequent hypocalcemia was temporarily related to zoledronic acid infusion, a well-documented complication in the literature, and was managed with IV and oral calcium, vitamin D, and calcitriol supplementation [7,16,18]. The management of HCM in adults is followed by guidelines set by the consensus from the Endocrine Society, which recommends IV bisphosphonates or denosumab as first-line treatment options and supports the use of calcitonin to reduce calcium in more severe cases [8]. In patients with contraindications to bisphosphonates, such as renal impairment or MRONJ in this case, denosumab is preferred, but its discontinuation requires careful planning and monitoring for rebound effects [8]. The use of zoledronic acid after denosumab withdrawal should be approached cautiously and closely monitored for hypocalcemia, especially in the weeks following infusion [7,16,18].

This case aligns with published reports describing the dual risks of rebound hypercalcemia after denosumab cessation and hypocalcemia following bisphosphonate therapy in patients with metastatic bone disease [7,9,10,16,18]. The higher incidence of MRONJ with denosumab, compared to zoledronic acid, is well established and signifies the need for preventive dental care and ongoing oral health surveillance throughout anti-resorptive therapy [4,5,13-15]. Optimal management of MRONJ and associated metabolic complications requires a comprehensive, collaborative approach between supportive care teams.

This case is limited by the absence of representative imaging, which would have supplemented the clinical and biochemical findings by directly visualizing bone destruction and pathological changes associated with MRONJ. The relevant imaging study was performed at an external institution, and we could not access the original CBCT imaging for inclusion due to interhospital electronic health record integration limitations. Despite our repeated efforts to obtain this data, this highlights the broader interoperability challenges in cross-institutional data sharing. While this limitation is notable, this report's clinical, biochemical, and narrative aspects continue to provide educational and clinical value.

## Conclusions

This study presents the complexity of managing calcium homeostasis in patients with MBC and bone involvement, particularly when anti-resorptive therapy is interrupted due to MRONJ. The patient’s clinical course displays the profound metabolic consequences that can arise during transitions in bone-targeted treatment, as demonstrated by severe rebound hypercalcemia following denosumab discontinuation and subsequent hypocalcemia after zoledronic acid initiation. Balancing adequate skeletal protection with the risk of treatment-related side effects remains a significant clinical challenge. Maximizing outcomes requires careful evaluation of dental health, identification of early metabolic imbalance, and cautious, individualized adjustments to therapy. This study contributes to the growing literature by demonstrating the need for patient-specific care to manage malignancy-associated bone disease, particularly for patients at risk for skeletal and metabolic complications.
